# How do seabirds modify their search behaviour when encountering fishing boats?

**DOI:** 10.1371/journal.pone.0222615

**Published:** 2019-09-24

**Authors:** Alexandre Corbeau, Julien Collet, Melissa Fontenille, Henri Weimerskirch

**Affiliations:** Centre d’Études Biologiques de Chizé, UMR7372 CNRS-La Rochelle Université, Villiers en Bois, France; Hawaii Pacific University, UNITED STATES

## Abstract

Seabirds are well known to be attracted by fishing boats to forage on offal and baits. We used recently developed loggers that record accurate GPS position and detect the presence of boats through their radar emissions to examine how albatrosses use Area Restricted Search (ARS) and if so, have specific ARS behaviours, when attending boats. As much as 78.5% of locations with a radar detection (contact with boat) during a trip occurred within ARS: 36.8% of all large-scale ARS (n = 212) and 14.7% of all small-scale ARS (n = 1476) were associated with the presence of a boat. During small-scale ARS, birds spent more time and had greater sinuosity during boat-associated ARS compared with other ARS that we considered natural. For, small-scale ARS associated with boats, those performed over shelves were longer in duration, had greater sinuosity, and birds spent more time sitting on water compared with oceanic ARS associated with boats. We also found that the proportion of small-scale ARS tend to be more frequently nested in larger-scale ARS was higher for birds associated with boats and that ARS behaviour differed between oceanic (tuna fisheries) and shelf-edge (mainly Patagonian toothfish fisheries) habitats. We suggest that, in seabird species attracted by boats, a significant amount of ARS behaviours are associated with boats, and that it is important to be able to separate ARS behaviours associated to boats from natural searching behaviours. Our study suggest that studying ARS characteristics should help attribute specific behaviours associated to the presence of boats and understand associated risks between fisheries.

## Introduction

Foraging behaviour is a central life-history trait because it determines energy acquisition [1]. When searching for resources, animals often display Area Restricted Search (ARS) behaviour whereby they increase sinuosity and reduce speed in specific areas [2–4]. This behaviour is generally assumed to increase the probability of encountering prey that are aggregated, patchy, and often widely dispersed [5]. Various methods have been developed to characterize ARS zones during movements of animals [6]. In marine or other environments where data on resource distribution is lacking, the ARS zones of predators have been used as proxies for areas of greater prey resource availability [7–9]. In the absence of better information on prey resource distribution, ARS zones of predators have also been used to define marine protected areas [10,11]. Seabirds are well-known to be attracted by fishing boats, and often forage behind these boats [12–14]. Seabirds can obtain important food resource from fishery offal or baits [15]. However, this food resource may be of poor nutritional quality [13] and fishery equipment such as long-lines and trawls can induce high seabird mortality [16]. Today the main threat for several seabird families, such as albatrosses and petrels, is the mortality induced by long-line fisheries [17,18].

During recent years, with the development of bio-logging techniques, it has become possible to study seabirds-fisheries interactions by combining tracking systems such as GPS and VMS (Vessel Monitoring System) data [19,20]. When interacting with fishing boats, seabirds often reduce their speed and alter their sinuosity, resulting in ARS behaviour [20,21]. When ARS zones are identified to determine foraging areas of seabirds, or to help designate marine protected areas, the occurrence of such interactions with fishing boats could lead to important and undesired biases. However, getting access to VMS or Automatic Identification System (AIS) data to quantify this bias is challenging for seabird researchers; access is often restricted for fisheries within national Exclusive Economic Zones (EEZs) and rarely exist or is incomplete for fisheries operating in international oceanic waters. Thus, when examining the movements and foraging behaviour of seabirds, it is difficult to attribute ARS movements to fishery presence or to the active search for natural resources.

Here we used recently developed loggers that record accurate GPS position and detect the presence of boats through their radar emissions [22] to examine whether albatrosses use ARS and have specific ARS behaviours, when attending boats compared with presumed natural foraging. Wandering albatrosses (*Diomedea exulans*) are strongly-attracted to fishing boats worldwide and are threatened by bycatch risks [18,23]. Previous tracking studies showed wandering albatrosses use ARS behaviour extensively at different spatial scales [24]. ARS behaviour, however, was not always associated with prey capture and it was not known whether ARS or prey capture were linked with the presence of a boat [24]. We hypothesized that 1) albatrosses should modify their ARS behaviour when attending boats, 2) the parameters describing the ARS (duration, sinuosity, and habitats) should be different from natural (not associated with a boat) ARS behaviour. We also examined whether ARS differed between habitats (shelves or oceanic waters) where different fisheries operate, to understand if behaviours and associated risks are influenced by the fishing types.

## Material and methods

Licences and permissions were granted by the Ethic Committee of Institut Polaire Francais (IPEV) and by the Préfet of Terres australes et antarctiques francaises (TAAF) after advices from the Comité de l'Environnement Polaire (CEP).

### Field work

**Fig 1:**
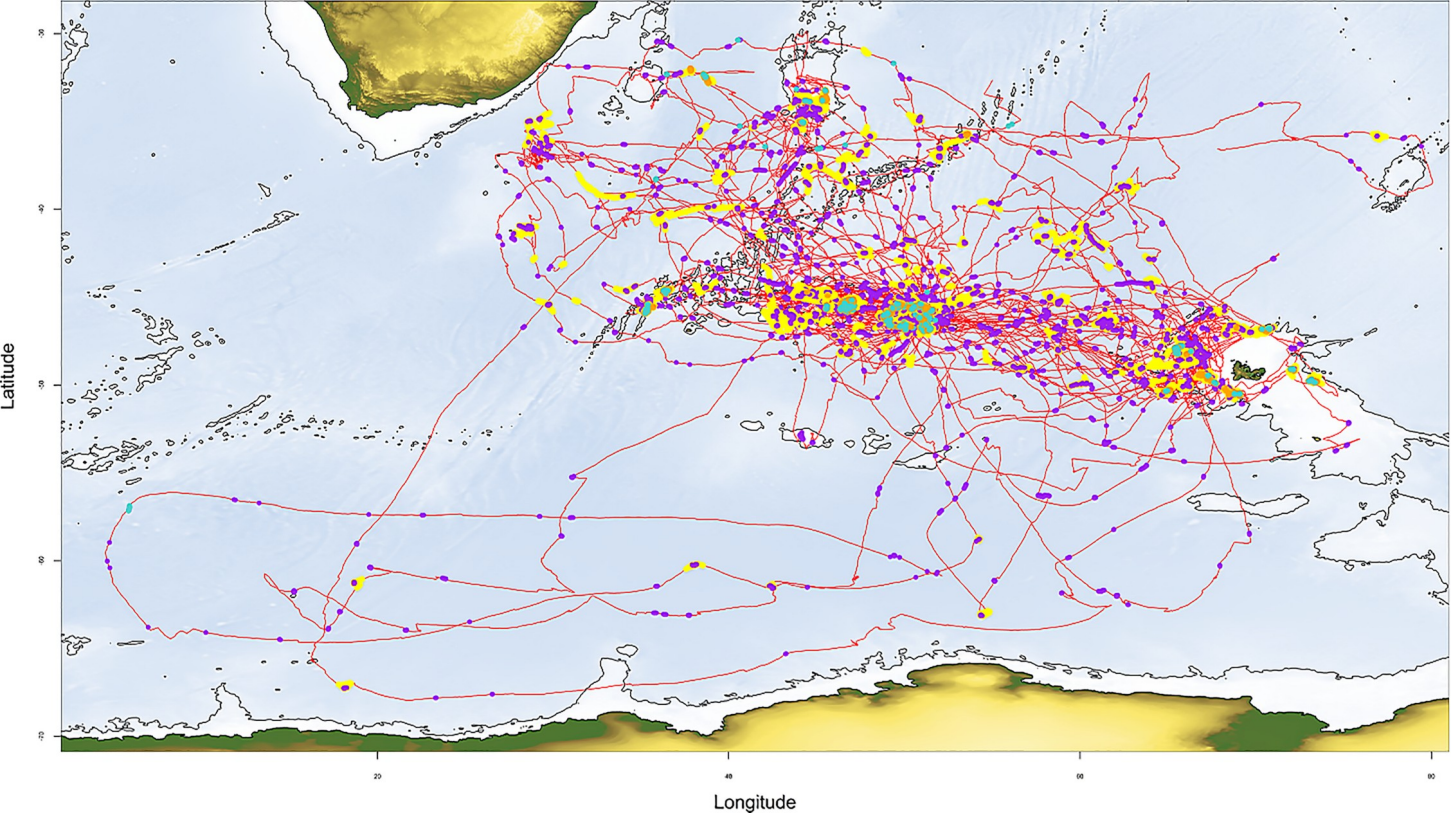
**Map of all wandering albatross trips (red).** ARS: one point per minute: yellow dot = large scales without radar detection and purple dot = small scales without radar detection; orange dot = large scales with radar detection; turquoise dot = small scales with radar detection. Bathymetry: isobaths for shelves (-2200 m deep).

The study was carried out on a population of wandering albatrosses from Possession Island, Crozet Islands (46°21’S; 51°42’E) during January–March 2016, 2017, and 2018. All wandering albatrosses Possession have been monitored annually from 1966 [23] and therefore all individuals are banded, sexed, and aged. The age of birds equipped ranged between 8 and 43 years. A total of 90 loggers (Fig 1) were deployed on 48 females and 42 males: 36 in 2016, 22 in 2017 and 32 in 2018.

Loggers (XGPS, Sextant technology–New Zealand) recorded GPS positions each minute and scanned for the presence of radar emissions (within 5 km maximum) for one minute each five minutes [22].

We affixed loggers on the back feathers with tape (Tesa® 4651, Beirersdorff, Germany) during a shift change with their partner, and each bird was weighed before release. When the bird returned to its nest after a foraging trip, the logger was recovered and the bird weighed again to estimate mass-gain during the foraging trip. Bird handling generally lasted less than 10 min, never exceeding 15 min. The mass of the logger was 60–75g (120x40x20mm), i.e. between 0.49% to 1.21% of the total weight of birds, much less than the 3% recommended for flying birds [25].

### Analysis

All data management and statistical analysis were performed under R environment (R Core Team 2017). We filtered data by removing all coordinates with speeds >100 km.h^-1^ [26].

#### Area restricted search

**Fig 2:**
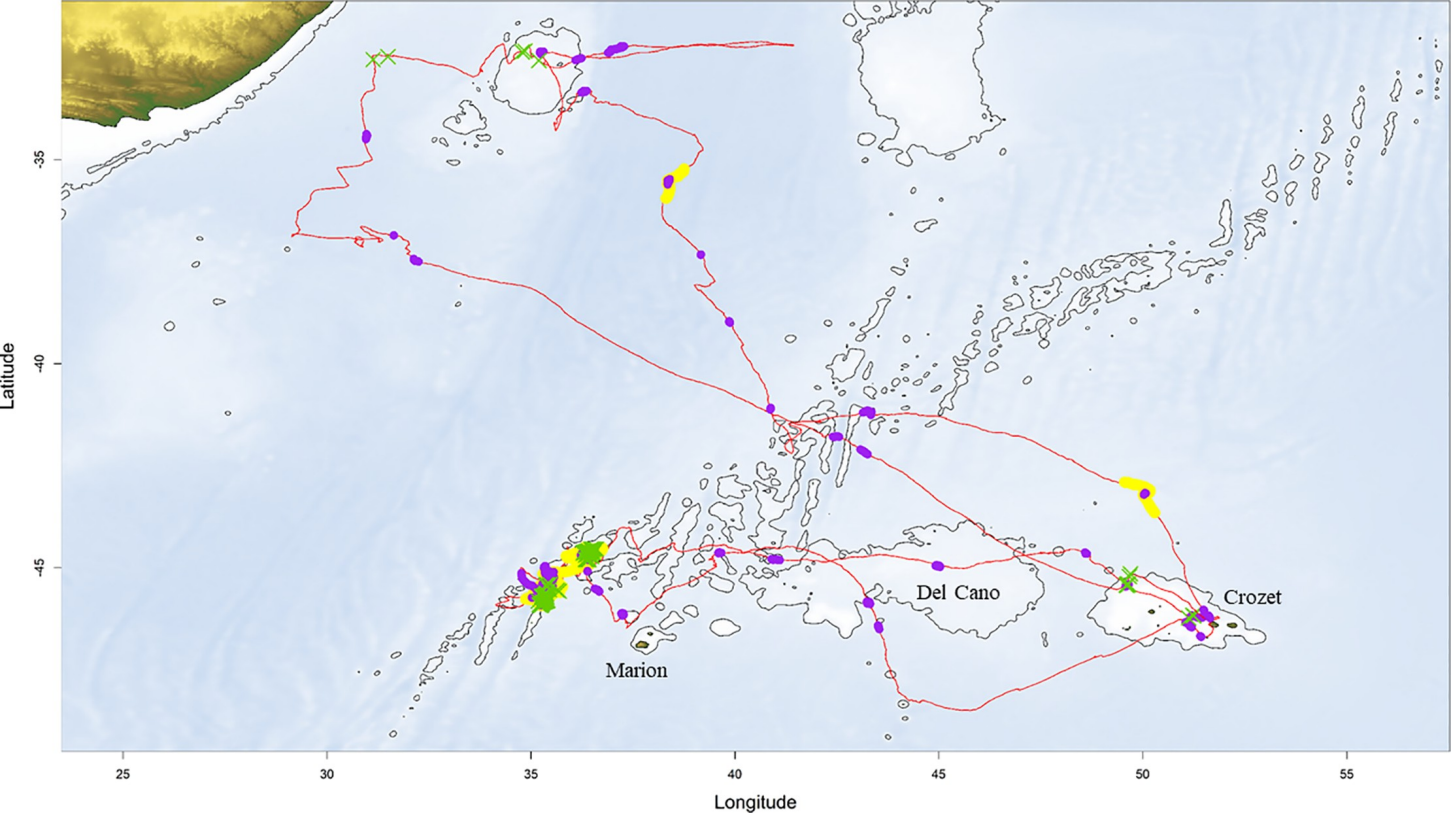
Map of two wandering albatross trips (red) with one over the shelf and one in oceanic waters. ARS: one point per minute: yellow dot = large scales; purple dot = small scales. Radar detections: green cross per location with boat detection. Bathymetry: isobaths for shelves (-2200 m deep).

Area-Restricted-Search (ARS) behaviours are performed at various scales [27], often with a nested structure (fine-scale intensive local search within a larger-scale ARS, Fig 2). To detect these ARS structures at multiple scales, we used the First Passage Time (FPT) method [28,29]. For ARS calculation, tracks were resampled with one location every segment of 1 km [27]. Because the standard variance peak procedure to identify putative scales of interest has been debated [30], we *a priori* fixed a range of 10 radius scales for analyses (kilometres): 2, 5, 10, 20, 30, 40, 60, 80, 100, 125, 150 km used in previous FPT analyses. Visual inspection and preliminary analyses led us to regroup them in three main categories for analyses: small scales (2, 5, 10 km), large scales (20, 40, 60, 80 km) and very-large scales (100, 125, 150 km). Because 83.2% of very-large scales ARS (n = 155) had nested, large-scale ARS and because analyses on very-large-scale ARS yielded very similar results to large-scale ARS, we report results only for small- (n = 1476) and large-scale ARS (n = 212) (Fig 1).

Wandering albatrosses are not active at night when they mainly sit on water to rest or sleep [31]. This can lead FPT analysis to spuriously identify night-resting areas as ARS [32]. We overcame this issue differently for small- and large-scale ARS. For small-scale ARS, we removed locations at night and only worked with daylight locations. For large-scale ARS, this was not possible because focusing our analyses on daytime only created border effects (of a size proportional to the scale of ARS investigated) where FPT cannot be calculated. Moreover some large-scale ARS can be performed over several consecutive days. Thus for large-scale ARS, we measured the proportion of the ARS duration that occurred at night, and included this measure in our analyses (see below) to control for its potential effect. Night-time was defined as the period when the sun was six-degree or more below the horizon (civil twilight).

We used R package ‘adehabitatLT’ [33] to calculate FPT values for each radius and then the Lavielle method [34,35] to segment the track based on their FPT values. Each segment was identified as ARS or not when its FPT values were above the average FPT value of the entire trip (large-scale ARS) or the average FPT value for the day (small-scale ARS).

#### Bathymetry data

We used the R package ‘marmap’ [36] to estimate depth values at each location (data extracted from ‘ETOPO1 Global Relief Model’ from ‘National Oceanic and Atmospheric Administration’). We estimated the average depth of each ARS, and classified ARS as either over oceanic waters (< -2200 m on average) or over shelves (> -2200 m). We also used it to created maps (Figs 1 and 2).

#### Descriptive parameters of ARS

For each ARS we calculated the following parameters: duration (h), sinuosity ($1-\frac{straight-line\;distance\;between\;the\;first\;and\;the\;last\;location\;of\;the\;ARS}{total\;distance\;travelled\;in\;the\;ARS}$), average distance to the colony (km), proportion of ARS during the night (large-scale ARS only), average depth (m), proportion of time spent sitting on water and for large ARS, the proportion of small ARS nested in larger ARS. We also estimated the proportion of time spent on the water during each ARS, by considering that birds were sitting on the water when speeds were <10 km.h^-1^ [26]. We considered that birds were associated with boats when at least one radar detection was recorded, knowing that loggers detect radar at a maximum distance of 5km [22]. We also considered an encounter as a series (from 1 to 220) of successive radar detections, with a least 2 hours with no radar detection between two encounters.

#### Statistical analyses

To analyse differences between natural ARS and boat-associated ARS, we used Generalised Linear Mixed Model (binomial family with individual included as a random factor) and estimated marginal R^2^ and conditional R^2^ following the Nakagawa & Schielzeth method (R package ‘MuMIn’ [37]) (Tables 1, 2 and 3). We analysed separately small- and large-scale ARS. Values are given as means ± one Standard Deviation, otherwise stated.

**Table 1:** Generalised linear mixed model results for differences between small/large scales of natural ARS and boat-associated ARS.

	Small scales					Large scales				
	Natural ARS (n = 1259)	Boat-associated ARS (n = 217) 14.702%	Differences (GLMM) (r^2^m = 0.398 r^2^c = 0.607)			Natural ARS (n = 134)	Boat-associated ARS (n = 78) 36.793%	Differences (GLMM) (r^2^m = 0.777 r^2^c = 0.906)		
	Mean ±sd	Mean ±sd	Slope	Z value	Significance	Mean ±sd	Mean ±sd	Slope	Z value	Significance
(Intercept)			-4.232	-8.731	***			-7.212	-2.042	*
Duration (h)	2.7 ±2.9	4.6 ±3.2	0.163	4.621	***	23.5 ±24.9	56.2 ±43.9	0.022	1.472	0.141
Sinuosity	0.5 ±0.3	0.7 ±0.2	3.600	7.277	***	0.6 ±0.2	0.9 ±0.1	11.444	2.661	**
Average distance to the colony (km)	772.5 ±656.6	538.2 ±571.1	-0.0005	-1.912	0.056	784.4 ±642.3	514.5 ±542.8	-0.001	-1.397	0.163
Average bathymetry (m)	-2626.3 ±1570.3	-1470.7 ±888.0	0.0006	5.214	***	-3086.3 ±1484.2	-1380.0 ±709.3	0.002	3.288	**
Proportion of time spent on water	0.5 ±0.3	0.6 ±0.2	-0.242	-0.563	0.573	0.5 ±0.2	0.6 ±0.1	3.945	1.659	0.097
Proportion of small ARS nested in larger ARS	0.3 ±0.4	0.7 ±0.5	1.105	4.949	***					
Proportion of night						0.5 ±0.3	0.4 ±0.1	-2.529	-1.314	0.189

Significant level

‘***’ <0.001

‘**’ <0.01

‘*’ <0.05.

**Table 2:** Generalised linear mixed model results for differences between small scales in ocean waters /over shelf of natural ARS and boat-associated ARS.

Small scales	Oceanic waters					Shelf				
	Natural ARS (n = 666)	Boat-associated ARS (n = 16) 2.40%	Differences (GLMM) (r^2^m = 0.010 r^2^c = 0.860)			Natural ARS (n = 593)	Boat-associated ARS (n = 201) 33.90%	Differences (GLMM) (r^2^m = 0.355 r^2^c = 0.573)		
	Mean ±sd	Mean ±sd	Slope	Z value	Significance	Mean ±sd	Mean ±sd	Slope	Z value	Significance
(Intercept)			-6.971	-2.640	**			-5.289	-8.881	***
Duration (h)	2.6 ±2.9	2.8 ±2.6	0.070	0.532	0.595	2.7 ±2.8	4.7 ±3.2	0.194	4.632	***
Sinuosity	0.5 ±0.3	0.5 ±0.2	-0.636	-0.447	0.655	0.6 ±0.2	0.8 ±0.2	4.318	7.388	***
Average distance to the colony (km)	990.8 ±681.1	1458.7 ±753.8	-0.0001	-0.228	0.820	527.2 ±529.9	464.9 ±486.3	-0.001	-3.139	**
Average bathymetry (m)	-3908.8 ±929.7	-3968.7 ±1062.2	0.0002	0.480	0.632	-1185.9 ±583.3	-1271.9 ±477.6	-0.0002	-0.923	0.356
Proportion of time spent on water	0.5 ±0.3	0.5 ±0.3	-0.327	-0.245	0.807	0.5 ±0.3	0.6 ±0.2	-0.394	-0.800	0.424
Proportion of small ARS nested in larger ARS	0.3 ±0.4	0.3 ±0.5	0.654	0.692	0.489	0.4 ±0.5	0.8 ±0.4	1.177	4.569	***

Significant level

‘***’ <0.001

‘**’ <0.01

‘*’ <0.05.

**Table 3:** Generalised linear mixed model results for differences between small scales ARS with boats detection on shelf or in oceanic waters.

Small scales boat-associated ARS	Oceanic waters (n = 16)	Shelf (n = 201)	Differences (GLMM)		
	Mean ±sd	Mean ±sd	Slope	Z value	Significance
(Intercept)			52.327	15034.100	***
Duration (h)	2.8 ±2.6	4.7 ±3.2	17.886	3132.400	***
Sinuosity	0.5 ±0.2	0.8 ±0.2	-12.547	-1812.900	***
Proportion of time spent on water	0.5 ±0.3	0.6 ±0.2	0.707	100.800	***
Proportion of small ARS nested in larger ARS	0.3 ±0.5	0.8 ±0.4	1.254	359.600	***

Significant level

‘***’ <0.001; ‘**’ <0.01; ‘*’ <0.05.

## Results

### Foraging trip characteristics

**Fig 3:**
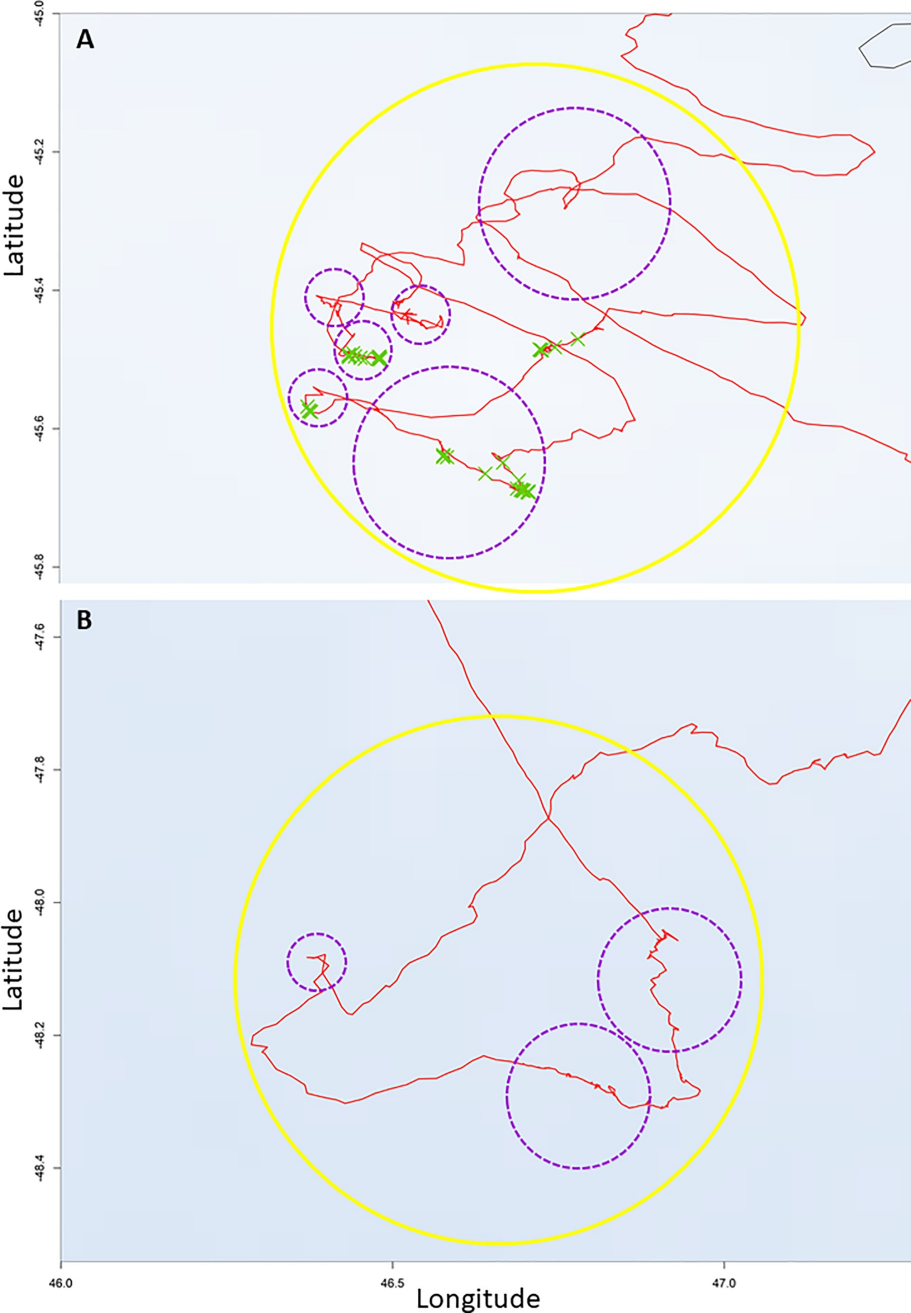
**Maps of boat-associated ARS (A)**: 1 large-scale ARS–solid yellow circle + 6 small-scale ARS nested–dotted purple circles) **and natural ARS (B)**: 1 large-scale ARS–solid yellow circle + 3 small-scale ARS nested–dotted purple circles)—Albatross trip (red line). Radar detections: green cross per location with radar detection. Bathymetry: isobaths for shelves (-2200 m deep).

Foraging trips consisted of rapid, direct movements interspersed with small and large ARS (Figs 1 and 2). There was no difference between males and females or among years in ARS characteristics (S1 Table), therefore we pooled sexes and years for analyses. All birds made small- and large-scale ARS. Of 90 birds tracked, 24 birds (26.7%) had no detection of radar during their trips (13 females and 11 males). On average, 78.5% (median = 96.9% and standard deviation = 33.8%) of locations with a radar detection during a trip (a contact with boats) occurred within an ARS identified with the FPT method (n _radar detection in ARS_ = 5386; n _all radar detection_ = 6368). During their trips, birds spent 22.2% ±7.3 of their time in small-scale ARS and 40.9% ±19.9 in large-scale ARS, 36.9% (n = 545) of small-scale ARS were nested in larger-scale ARS (Fig 3). Birds spent 0.9% ± 1.3% of their total foraging time directly associated (with at least a radar detection) with a boat and had on average 4.3 ± 4.8 boat encounters per trip (maximum = 21). We considered ARS without radar detection to be ‘natural’ ARS (n = 1393), and those with radar detections to be ‘boat-associated’ ARS (n = 295).

### Difference between natural ARS and boat-associated ARS

Only 14.7% of small-scale ARS (n _all small ARS_ = 1476) and 36.8% of large-scale ARS (n _all large ARS_ = 212), 36.8% were associated with the presence of a boat.

**Fig 4:**
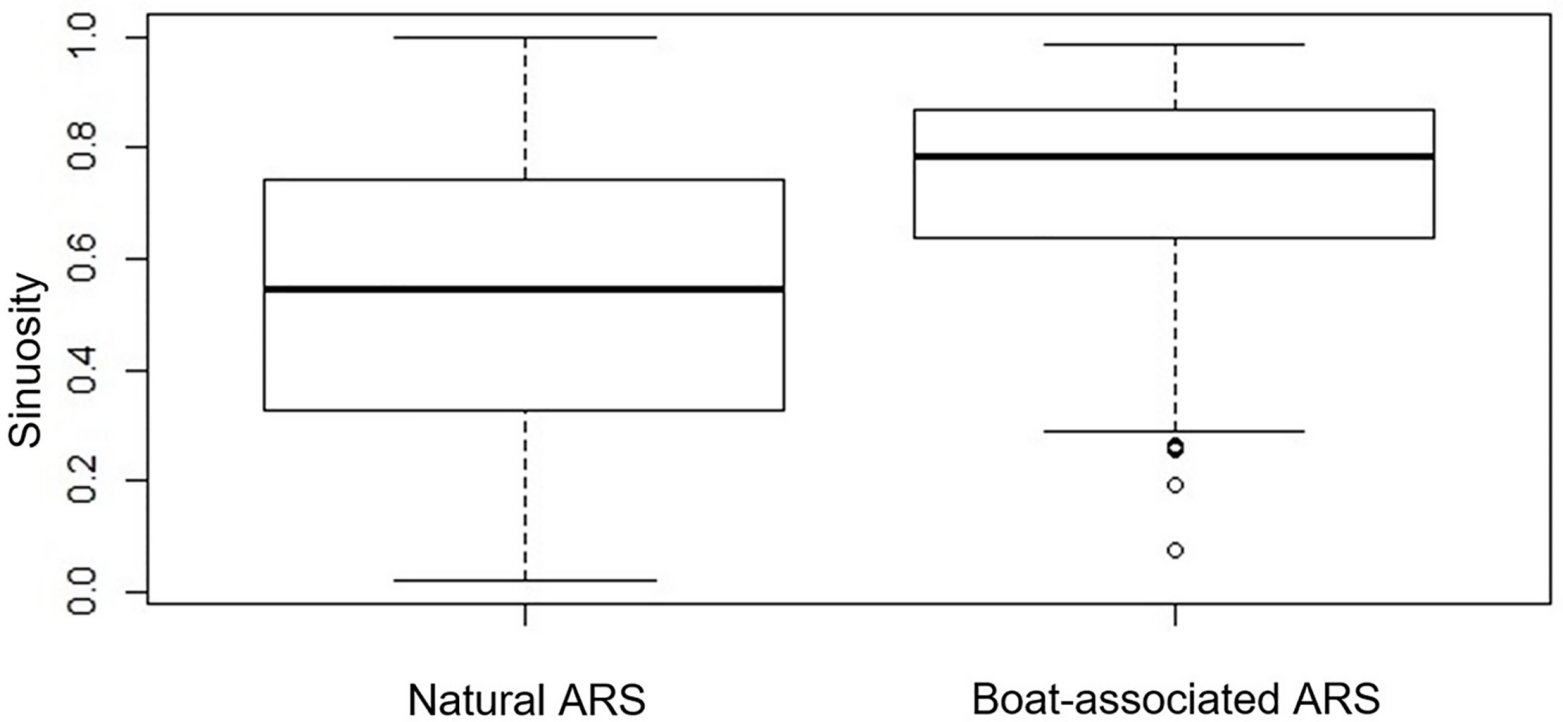
Sinuosity boxplot of small scales ARS: Natural ARS are significantly different than ARS associated with a boat.

For small-scale ARS, birds spent more time and had greater sinuosity during boat-associated ARS compared to natural ARS (Figs 3 and 4, Table 1 –Small-scale ARS, GLMM: marginal R^2^ = 0.398 and conditional R^2^ = 0.607; Y = -4.232 + 0.163 duration of ARS + 3.6 sinuosity of ARS—0.0005 average distance to the colony of the ARS + 0.0006 average bathymetry of ARS -0.242 proportion of time spent sitting on water in ARS). Small-scale, boat-associated ARS were performed over shallower waters and mostly occurred over shelves (Table 2) and tended to be more frequently nested in larger-scale ARS (Table 1) than natural ARS. There was no significant difference in the proportion of time spent sitting on water and in the average distance to the colony between natural and boat-associated small ARS (Table 1 –Small scales).

For large-scale ARS, boat-associated ARS were more sinuous than natural ARS (Fig 3), but there was no difference in duration, average distance to the colony, and proportion of time spent sitting on water between natural and boat-associated ARS. For large-scale ARS, there was no difference in the proportion of night spent between natural and boat-associated ARS (Table 1 –Large scales).

### Difference between oceanic and shelves small-scale ARS

When comparing small-scale ARS performed over oceanic waters from those over shelves, we found no significant differences in the associated parameters between natural and boat-associated ARS in oceanic waters (Table 2 –Oceanic waters). For small scale ARS over shelves, duration, sinuosity and proportion of small ARS nested in larger ARS were higher for boat-associated ARS compared to natural. ARS with boats were also on average closer to the colony than natural ARS (Table 2 –Shelf).

If we consider only ARS associated with boats, small-scale ARS over shelves were longer in duration, had greater sinuosity, and birds spent more time sitting on water compared with oceanic ARS (Table 3).

## Discussion

Our study is the first to precisely estimate to what extent ARS behaviours in seabirds are associated with the presence of boats. Previous studies showed that the behaviour of birds associated with boats equipped with VMS had different behaviour from other ARS [20,21,38], that could be natural or associated with boats without VMS. By using loggers equipped with radar detectors, we have shown that as much as 78% of boat detections occurred in an ARS, and that wandering albatrosses, modified their movements when associating with boats. Albatrosses are attracted by boats and may associate with them for variable durations; they can either have a brief encounter lasting a few minutes while following a cruising boat, or they may attend a fishing boat in operation [22,39]. In the latter case, birds probably entered into an ARS behaviour, whereas for the first cases, they do not probably use ARS. However, our results showed that the majority of ARS were not associated with boats, and thus this searching behaviour can be considered a natural foraging behaviour.

When attending boats, ARS had different characteristics from natural ARS, they were longer in duration, more sinuous, and occurred over shallower waters. Since these ARS probably occurred with fishing boats, birds may have stayed for long periods behind boats to access food, waiting for the release of offal or the setting of long–lines when they try to take baits. Greater sinuosity may be explained by the specific movement of birds whereby they continuously take-off and land using wind to stay close from the boats in operation, but also when they followed a slow moving fishing boat in operation. At finer temporal scales (<30 min), sinuosity can be lower if albatrosses following fishing boats, and particularly long-liners, may actually display locally very straight paths as a fishing line is set or hauled [20], but our results showed that this did not occur during natural or boat-associated ARS.

We also found that a large proportion of small-scale ARS were nested in larger-scale ARS when birds were associated with boats, compared to natural ARS. This could reflect the movements of fishing boats themselves, but most of the duration of large-scale ARS were not associated with fishing boats. We suggest nested ARS structure arises from a common large-scale habitat selection between boats and albatrosses. A large proportion (57%) of larger-scale ARS occurred over shelf-edges or seamounts (e.g. south of Madagascar), where many fishing boats operate. Wandering albatrosses may recognise the boundaries of these areas and increase their search intensity over these shallower waters [40–42]. Indeed it was shown previously that ARS in this species were not necessarily triggered by prey capture [9], but high-foraging efficiency could also be achieved if they were triggered by favourable habitat recognition [5,43]. Then, when encountering and interacting with fishing boats in these areas, they would display finer-scale, nested ARS behaviour. This would also explain why boat-associated ARS were more likely to be nested when over shelves compared with oceanic waters.

In the southern Indian Ocean, fisheries operate either over shelves (and especially shelf-edges) or over oceanic waters. In oceanic waters, in the range of wandering albatrosses, extensive long-line fisheries operate in sub-tropical and tropical waters where they target various species of tuna and cause high albatross mortality [17,23,44]. Over shelf-edges or shelves, Crozet wandering albatrosses encounter predominately long-liners targeting Patagonian tooth-fish around Crozet and Kerguelen Islands and other shelves in sub-Antarctic waters such as the Del Cano rise (Fig 2). These fisheries also caused high mortality historically, but now that they are regulated in EEZs, these fisheries have reduced albatross mortality in EEZs [18]. In subtropical waters, wandering albatrosses encounter fishing boats over seamounts, especially south of Madagascar (Fig 2), but also over oceanic waters. ARS behaviours associated with boats probably occur mostly with fishing boats in operation [39]. We found that ARS behaviour differed between oceanic-tuna fisheries and shelf-edge fisheries (mainly Patagonian toothfish). Over shelf edges, small-scale ARS were longer in duration and more sinuous, indicating more intense foraging behaviour compared to oceanic-tuna fisheries. These differences may be due to different operational practices between these fisheries, longer-lines (thus duration of line-setting and hauling), and different baits and offal [45].

This study showed that a significant proportion (21.2%) of ARS behaviours made by wandering albatrosses occurred in association with boats and that 73% of birds encountered a boat during their foraging trips. Our results demonstrate fisheries can extensively modify the foraging behaviour of seabirds such as albatrosses. Natural ARS behaviour, however, remains by far the majority of the foraging behaviour. Because long-line fisheries induce high mortality of albatrosses, it is important to be able to determine whether foraging birds associate with a boats and increase risk. Our study constitutes an important and promising step towards accurate quantitative predictions of vessel association at sea. Developing predictive analyses through unsupervised machine learning approaches [46] or by the use of Hidden-Markov-Model (HMM) [47,48] should allow scientists to determine the degree to which movement recorded simply by GPS, may be related to the presence of a boat. Having access to this predictive capability could open up interesting perspectives on retrospective studies with tracking data and how the attraction of albatrosses to boats may have ‘evolved’ throughout decades of GPS tracking [49].

## Supporting information

S1 Table Differences between females and males for trips and ARS parameters. Significant level: ‘***’ <0.001; ‘**’ <0.01; ‘*’ <0.05.
(DOCX)
