# Supplementary material for: How do seabirds modify their search behaviour when encountering fishing boats?
Source: PLoS One. 2019 Sep 24;14(9):e0222615. doi: 10.1371/journal.pone.0222615 (PMC6759163; doi:10.1371/journal.pone.0222615)
Supplement: S1 Table — Significant level: ‘***’ <0.001; ‘**’ <0.01; ‘*’ <0.05. (DOCX) [file pone.0222615.s001.docx]

**Supporting information**

**S1 Table – Differences between females and males for trips and ARS parameters.**

|  | **Mean** | **Standard deviation** | **Mean** | **Standard deviation** | **Z value** | **Significance (P value)** |
| --- | --- | --- | --- | --- | --- | --- |
| **Parameters for trip** | | | | | | |
|  | **Female (n=48)** | | **Male (n=42)** | | **Difference** | |
| Proportion of radar detection who are in ARS | 0.732 | 0.366 | 0.846 | 0.265 | 1.203 | 0.229 |
| Time spent behind boat (h) | 17.368 | 18.689 | 17.543 | 16.190 | 1.276 | 0.202 |
| Maximum range (km) | 1081.400 | 718.676 | 1074.400 | 795.702 | 1.104 | 0.270 |
| Proportion of radar | 0.008 | 0.011 | 0.010 | 0.016 | -0.695 | 0.487 |
| Proportion of small ARS | 0.204 | 0.068 | 0.241 | 0.074 | 2.534 | * |
| Proportion of medium ARS | 0.408 | 0.198 | 0.409 | 0.203 | -0.020 | 0.984 |
| Proportion of large ARS | 0.377 | 0.217 | 0.361 | 0.197 | 0.591 | 0.555 |
| Total distance (km) | 5373.900 | 2965.777 | 4916.800 | 3454.978 | -0.721 | 0.471 |
| Average speed (km.h-1) | 22.540 | 6.383 | 21.301 | 7.034 | 1.722 | 0.085 |
| Average bathymetry (m) | -2453.800 | 960.523 | -2332.200 | 903.936 | 1.747 | 0.081 |
| Proportion of time spent on water | 0.4243 | 0.124 | 0.457 | 0.101 | 1.904 | 0.057 |
| Proportion of small ARS nest in large ARS | 0.104 | 0.079 | 0.115 | 0.078 | -1.370 | 0.171 |
| Proportion of medium ARS nest in large ARS | 0.120 | 0.073 | 0.138 | 0.096 | -0.581 | 0.561 |
| Proportion of small ARS nest in medium ARS | 0.307 | 0.188 | 0.281 | 0.172 | -0.644 | 0.520 |
| Number of boats encounter | 4.688 | 5.684 | 3.880 | 3.514 | -1.309 | 0.191 |
| **Parameters for ARS** | | | | | | |
|  | **Female (n=1030)** | | **Male (n=813)** | | **Difference** | |
| Duration (h) | 12.960 | 28.748 | 13.280 | 25.093 | 0.249 | 0.803 |
| Proportion of radar detected | 0.057 | 0.165 | 0.071 | 0.189 | -0.123 | 0.902 |
| Total distance (km) | 160.430 | 423.737 | 133.600 | 269.563 | -0.284 | 0.777 |
| average speed (km.h-1) | 13.920 | 10.777 | 13.420 | 12.083 | 0.148 | 0.883 |
| sinuosity | 0.594 | 0.259 | 0.612 | 0.254 | 0.189 | 0.850 |
| average distance to the colony (km) | 710.400 | 604.480 | 728.940 | 690.644 | -0.084 | 0.933 |
| proportion of night | 0.098 | 0.205 | 0.100 | 0.201 | -0.071 | 0.944 |
| average bathymetry (m) | -2513.000 | 1454.600 | -2306.000 | 1598.929 | 0.361 | 0.718 |
| proportion of time spent on water | 0.512 | 0.248 | 0.546 | 0.239 | 0.256 | 0.798 |
| proportion of small ARS nest in large ARS | 0.257 | 0.437 | 0.246 | 0.431 | -0.168 | 0.867 |
| proportion of medium ARS nest in large ARS | 0.0714 | 0.254 | 0.074 | 0.260 | -0.076 | 0.940 |
| proportion of small ARS nest in medium ARS | 0.278 | 0.447 | 0.259 | 0.437 | -0.015 | 0.988 |
| time spent in contact to boat (h) | 0.105 | 0.344 | 0.150 | 0.386 | 0.202 | 0.840 |
| distance from the beginning to the end of ARS (km) | 28.460 | 54.290 | 26.148 | 49.562 | 0.105 | 0.916 |

Significant level: ‘***’ <0.001; ‘**’ <0.01; ‘*’ <0.05.
